# FaSmi1 Is Essential for the Vegetative Development, Asexual Reproduction, DON Production and Virulence of *Fusarium asiaticum*

**DOI:** 10.3390/jof8111189

**Published:** 2022-11-11

**Authors:** Yu Zhang, Wenchan Chen, Wenyong Shao, Shishan Tan, Dongya Shi, Hongyu Ma, Changjun Chen

**Affiliations:** 1 The Key Laboratory for Quality Improvement of Agricultural Products of Zhejiang Province, Department of Plant Pathology, College of Advanced Agricultural Sciences, Zhejiang A&F University, Hangzhou 311300, China; 2 The Key Laboratory of Pesticide, College of Plant Protection, Nanjing Agricultural University, Nanjing 210095, China; 3 Nanjing Pukou District Agricultural Technology Extension Center, Nanjing 211800, China

**Keywords:** *Fusarium* head blight, *Fusarium asiaticum*, FaSmi1, virulence

## Abstract

Smi1 is a protein required for cell cycle progression, morphogenesis, stress response and life span of *Saccharomyces cerevisiae*. FaSmi1 was identified as a Smi1 homolog in a wheat scab pathogenic fungus *Fusarium asiaticum* strain 2021. The deletion of FaSmi1 leads to defects in mycelial growth, asexual reproduction, and virulence. The FaSmi1 deletion mutant also exhibited increased sensitivity to osmotic stresses generated by NaCl and KCl, but increased tolerance to oxidative stresses and cell wall integrity inhibitors. All of these defects were restored by genetic complementation of the mutant with the whole parental *FaSmi1* gene. Interestingly, the antioxidant system-associated genes exhibit a lower expression level and the mycotoxins’ DON content was decreased in the FaSmi1 deletion mutant compared with the parental strain 2021. These results indicate that FaSmi1 plays a critical role in the vegetative development, asexual reproduction, DON production and virulence of *F. asiaticum*.

## 1. Introduction

*Fusarium* head blight (FHB) caused by the *Fusaruim* complex species is a destructive plant disease and leads to large economic damage around the world. FHB quickly spreads in regions with high temperatures and humidity, causing 10–50% cereal yield loss, and the mycotoxins produced by *Fusarium* complex species in the infected wheat and other cereal crops pose a serious threat to human and animal health [1,2]. Despite the large economic impact of FHB, the main strategy for controlling FHB is the application of fungicides [3]. Only a few kinds of fungicides (including carbendazim azoxystrobin and triazoles) are available to reduce the FHB index, but they cannot control the accumulation of deoxynivalenol (DON) in cereals. Moreover, on account of the frequent and large application of fungicides to control FHB, the *Fusarium* complex species have developed resistance against various fungicides, leading to a weaker control effect on FHB and even failure [4,5]. Based on the current situation of FHB control, the exploitation of new types of inhibitors for the management of FHB is needed. Therefore, we explored key proteins required for vegetative development, DON production and virulence in *Fusarium* complex species, which might be considered a potential target protein for developing new fungicides to control FHB.

Smi1, also known as Knr4, is an intrinsically disordered protein conserved in many fungi [6]. In *Saccharomyces cerevisiae*, Smi1 plays important roles in the Slt2 MAP kinase cell wall integrity pathway and the calcineurin phosphatase in the calcium–calcineurin pathway via physically interacting with the key components of two pathways [6,7]. Smi1 has numerous functional interaction partners, leading to synthetic lethal interactions [8,9]. In various fungi, Smi1 is involved in the cell cycle, morphogenesis and stress response by regulating associated transcriptional programs [10,11,12]. In budding yeast, the deletion of Smi1 caused the transcription factor SBF to be constitutively hyperactivated rather than to peak at the G1/S transition, leading to defects in coordinating cell division with bud growth [13,14,15,16] and in the mechanism regulating the size of daughter cells [7]. Smi1 homologs play similar roles in the polarized growth of other fungi [10]. Smi1 is required for the nuclear accumulation of Msn2 and enhances the transcription of PNC1, which is responsible for extending the replicative life span and Sir2-mediated rDNA stability in *S. cerevisiae* [17]. In addition, Smi1 is involved in efficient agrobacterium-mediated yeast transformation with chromosomal T-DNA [16,18]. From these reports, we considered that Smil may play various important functions in different fungi, especially in yeast. Therefore, we hypothesized that the Smi1 homolog plays important roles in *Fusarium* complex species. 

In this study, we identified the *S. cerevisiae* Smi1 homologous protein, named FaSmi1 in *F. asiaticum*, to be an important plant pathogen to *Fusarium* complex species. We investigated the biological roles of FaSmi1 in *F. asiaticum* via generating a FaSmi1 deletion mutant and analyzed its phenotypes. Our results show that FaSmi1 is a key protein required for the vegetative development, asexual reproduction, DON production and virulence of *F. asiaticum*. Therefore, we propose that FaSmi1 could be considered a potential target protein for developing new fungicides to control FHB caused by *F. asiaticum*. These results provide valuable information for better FHB control strategies. 

## 2. Materials and Methods

### 2.1. Strains and Culture Conditions 

*F. asiaticum* strain 2021 was originally isolated from infected wheat ears in Zhejiang province of China. It was used as the wild-type strain to generate gene deletion mutants. Potato dextrose agar (PDA), complete medium (CM) and minimal medium (MM) were used for mycelia growth and stress sensitivity assays [19,20]. Mung bean liquid (MBL) liquid medium was used for conidiation assays [21]. Liquid trichothecene biosynthesis (LTB) liquid medium was used for DON production assays [22].

### 2.2. Sequence Analysis of FaSmi1 in F. asiaticum

FaSmi1 is a homologous protein of FgSmi1 (The accession number in *Fusarium graminearum* genome FGSG_06998.3) that was originally identified through BLASTP searches in the *F. graminearum* genome (available at http://www.broadinstitute.org/annotation/genome/fusarium_group/Multi-Home.html, accessed on 16 March 2018), referring to the Smi1 from *S. cerevisiae*. Based on the sequence information of the *FgSmi1* gene, full-length and cDNA of *FaSmi1* was amplified from genomic DNA of wild-type strain 2021 for the sequence analysis.

### 2.3. Generation of FaSmi1 Deletion Mutants

To explore the roles of FaSmi1 in *F. asiaticum*, we generated *FaSmi1* deletion mutants via the homology replacement method, as described previously [23]. Briefly, the gene replacement cassette was constructed, which carries the hygromycin resistance gene and herpes simplex virus thymidine kinase gene flanked by the 5′ (upstream junction) and 3′ (downstream junction) ends of the *FaSmi1* gene. This cassette was constructed with double-joint PCR, as previously described [24]. The fragments 1.3 kb upstream and 1.2 kb downstream of *FaSmi1* were amplified from the genomic DNA of strain 2021 using the primer pairs P1/P2 and P3/P4, respectively. The 3.5-kb fragment with trpC promoter of *A. nidulans*, hygromycin resistance gene and the thymidine kinase gene from the herpes simplex virus and the (HPH-tk) was amplified from pKHT plasmid using the primer pair HTF/HTR [23] (Table S1). After the abovementioned three fragments (up and downstream of FaSmi1 and HPH-tk) were purified by a gel purification kit, which was mixed with a molar ratio of 1:3:1 and used as a template to perform a double-joint PCR. After the fragment was amplified by PCR using 5 μL product of a double-joint PCR and primers P5/P6 (Table S1), the PCR product was purified and sequenced. Next, after the sequencing result was confirmed and the PCR product was transformed into protoplasts of *F. asiaticum* strain 2021 to generate *FaSmi1* deletion mutant. 

### 2.4. Complementation of FaSmi1 Deletion Mutants 

The *FaSmi1* deletion mutant (Δ*FaSmi1*) was complemented with the full-length *FaSmi1* gene to confirm that the phenotypic changes of the *FaSmi1* deletion mutant were due to the disruption of the gene. The construct for the complementation of Δ*FaSmi1* was generated by amplifying a fragment from the genomic DNA of strain 2021 with primer P1/P4 (Table S1). After being purified and sequenced, the PCR product was transformed into protoplasts of Δ*FaSmi1*. 

### 2.5. Protoplast Preparation and Transformation of F. asiaticum

For preparation of protoplasts, conidia of the 2021 strain were harvested from seven-day-old cultures grown in MBL medium and inoculated into YEPD liquid medium (10 g peptone, 3 g yeast extract, 2 g glucose per 1 L ddH_2_O). Mycelia were isolated via gauze filtration after incubation at 175 rpm and 25 °C for 12 h. Then, mycelia were washed with 0.7 M NaCl buffer and incubated with lysing buffer (0.2 g lysing, 0.2 g helicase and 0.1 g driselase dissolved in 20 mL 0.7 M NaCl), and the protoplasts were isolated via gauze filtration after incubation at 75 rpm and 30 °C for 2 h. The protoplasts were washed twice using 0.7 M NaCl and STC (50 mM Tris pH 8.0, 0.8 M sorbitol, 0.05 M CaCl_2_), respectively, then resuspended in STC-SPTC buffer [STC:SPTC = 4:1; SPTC (STC containing 40% PEG 6000)]. In the transformation, protoplasts (10^7^ cells /mL), 3 mg target DNA and heparin sodium were added into 200 μL SPTC buffer and were mixed. After incubation for 30 min on ice, 1 mL SPTC was added and mixed, continuing incubation for 20 min at room temperature. Next, transformed protoplasts were added into 200 mL RM medium (274 g sucrose, 1 g yeast extract, 1 g casein hydrolyzate, 17 g agar, per 1 L ddH_2_O) at 43 °C. After sufficient mixing, RM medium containing protoplasts was poured into 9 cm diameter culture plates (15 mL per plate) and incubated at 25 °C for 12 to 16 h. Then, the plates were overlaid with 10 mL of SRM medium (342 g sucrose, 1 g yeast extract, 1 g casein hydrolyzate, 10 g agarose, per 1 L ddH_2_O) modified with 100 μg/mL hygromycin B. Transformants were obtained after 3–5-day incubation at 25 °C and were transferred onto PDA plates supplemented with 100 μg/mL hygromycin B (but complementation strains could not grow) and 0.2 μM floxuridine (but transformants could not grow). Complementation of Δ*FaSmi1* with the wild-type *FaSmi1* gene was performed as described above, except the selection agent was floxuridine.

### 2.6. Mycelial Growth, Conidiation and Stress Sensitivity Assay

The mycelial growth assay was conducted on PDA. Mycelial plugs of each strain taken from the margin of the colony were put onto PDA. The colony diameter of each strain was measured after incubation for 3 days at 25 °C in the dark. Each treatment had three replicates; the experiment was repeated three times.

We analyzed the sensitivity of the deletion mutant to osmotic stress, which was generated by NaCl and KCl, and cell wall integrity inhibitors (Congo red and caffeine), and cell membrane damage generated by SDS. The mycelial plugs of each strain were incubated on PDA plates supplemented with 1.2 M NaCl, 1.2 M KCl, 0.05% Congo red, 5 mM caffeine, and 0.05% SDS for 3 d at 25 °C in the dark. The relative inhibition growth rate was calculated by the following formula: [(A − B)/(A − 5)] × 100, where A and B are the colony diameter of control and treatment, respectively [25]. Each treatment had three replicates; the experiment was repeated three times.

In conidiation assay, eight mycelial plugs of each strain were put onto 250 mL broth containing 100 mL MBL medium incubated at 25 °C with shaking at 175 rpm for a 16-h photoperiod. After seven days, spores were counted with a hemocytometer [26]. Each treatment had three replicates; the experiment was repeated three times.

### 2.7. Quantitative RT-PCR (qRT-PCR)

The total RNA of each sample was isolated with a total RNA isolation Kit (Invitrogen, Carlsbad, CA, USA). First-strand cDNA was synthesized with the PrimeScript^®^ RT reagent kit (TaKaRa, Kusatsu, Japan). All qRT-PCR reactions were performed with a qRT-PCR assay Kit (Vazyme, Nanjing, China) and ABI 7500 real-time detection system (Applied Biosystems, Waltham, MA, USA). Primers used for qRT-PCR analysis are listed in Table S1. All data were normalized to actin gene expression, and relative changes in gene expression levels were analyzed with ABI 7500 SDS software (Applied Biosystems), which automatically set the baseline. Data from three biological replicates were used to calculate the means and standard deviations. The experiment was repeated three times.

### 2.8. Virulence Assay on Flowering Wheat Heads

After incubation in MBL medium for 7 days, conidia of each strain were collected by filtration through three layers of lens paper and subsequently resuspended in sterile distilled water and adjusted to a concentration of 10^6^ conidia/mL. Wheat heads of the Zhenmai 22 cultivar were inoculated with 10 μL of a conidial suspension of each strain, as described by Gale [27]. After being moisturized for 2 days, the wheat plants were cultivated in a greenhouse. Each treatment had 20 wheat heads. After 15-day inoculation, the number of infected spikelets in each treatment was measured, and the data were assessed with the Fisher’s LSD test. The experiment was repeated three times.

### 2.9. In Vitro DON Production Assay

For the in vitro total DON production analysis, conidia of each strain were inoculated into LTB liquid media (1 × 10^5^ conidia/mL). The total DON production of each strain in LTB cultures was assayed with a competitive ELISA-based DON detection plate kit (Beacon Analytical Systems, Inc., Saco, ME, USA) after incubation at 28 °C for 7 days, as described [22]. The total DON production ability (DPA) of each strain was calculated by the formula: DPA = total DON production in LTB cultures (mg).

## 3. Results

### 3.1. Identification of FaSmi1 in F. asiaticum

Using BLASTP, FaSmi1 was identified in the *F. asiaticum* genome, based on amino acid sequences of Smi1 in *S. cerevisiae* and *F. graminearum* genome data. FaSmi1 is highly homologous to its counterparts among various fungal species (Figure S1). The sequencing data indicated that, in *F. asiaticum*, full-length *FaSmi1* is 1678 bp, containing two introns, coding 571 amino acids, and the structural domain analysis showed that FaSmi1 has a SMI1/KNR4-conserved domain which is involved in the synthesis of β-1,3 glucan and cell wall integrity. 

### 3.2. Deletion and Complementation of FaSmi1 in F. asiaticum

To investigate the functions of FaSmi1 in *F. asiaticum*, we generated the *FaSmi1* deletion mutant (Δ*FaSmi1*) by transforming the gene replacement construct containing an *HPH-tk* resistance cassette into the parental strain 2021 (Figure S2A). The FaSmi1 deletion mutant was identified by PCR and southern blotting using special primer pairs and a probe, respectively (Figure S2B,C). To ensure the phenotypes of deletion mutants were caused by deleting the corresponding gene, the *FaSmi1* deletion mutant was complemented with the parental *FaSmi1*. The complementation strain (Δ*FaSmi1C*) was confirmed by southern blotting (Figure S2C).

### 3.3. Involvement of FaSmi1 in Hyphal Growth and Asexual Development of F. asiaticum

**Figure 1:**
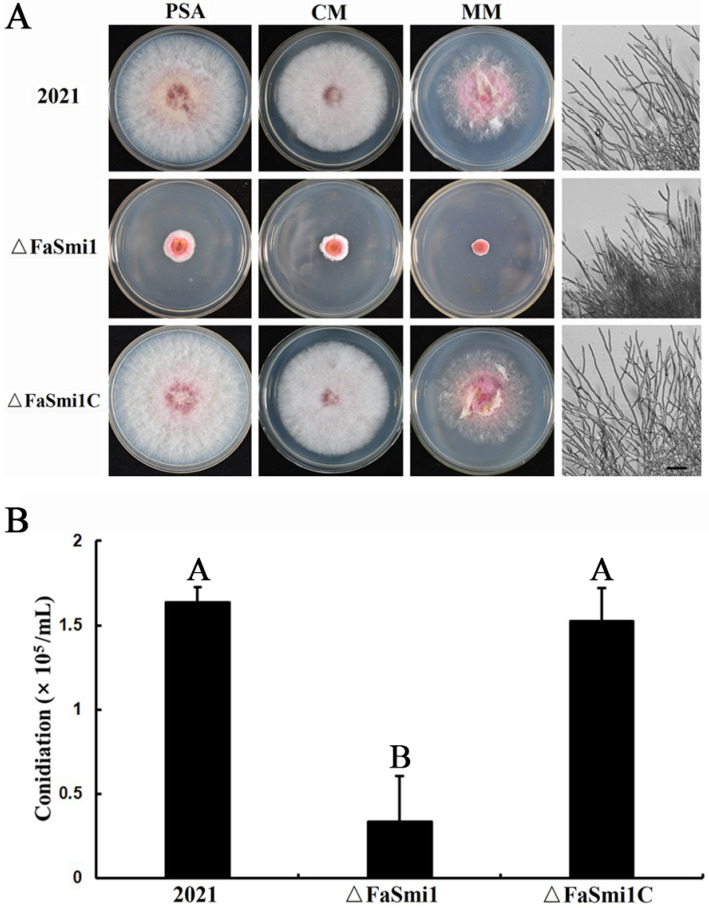
Deletion of FaSmi1 effect on mycelial growth and conidiation. (**A**) The colony microscopic assay of 2021, Δ*FaSmi1* and Δ*FaSmi1C* was photographed after incubation on PDA, MM and CM for 3 days. Bar = 12 μm. (**B**) Mycelial plugs of 2021, Δ*FaSmi1* and Δ*FaSmi1C* were grown on MBB at 25 °C for 7 days; the conidiation of each strain were measured. Bars denote standard errors from three experiments. Values on the bars followed by the same letter are not significantly different at *p* = 0.05.

Compared to the parental 2021 strains, the mycelial growth rate of Δ*FaSmi1* was decreased (Figure 1A; Table 1) and the pink pigment production of Δ*FaSmi1* was increased on PDA media (Figure 1A). To explore whether the mycelial growth defects were associated with the medium, we incubated the 2021, Δ*FaSmi1* and Δ*FaSmiC* strains on PDA, CM and MM media. Compared to 2021 and Δ*FaSmi1C*, the mycelial growth defects of Δ*FaSmi1* were also observed on CM and MM medium (Figure 1A; Table 1). Moreover, in the microscopic assay, the mycelia of Δ*FaSmi1* were thicker and had thinner branches compared to 2021 and Δ*FaSmi*C (Figure 1A). These results indicate that *FaSmi1* plays a significant role in the mycelial growth and pigment generation of *F. asiaticum*. 

**Figure 2:**
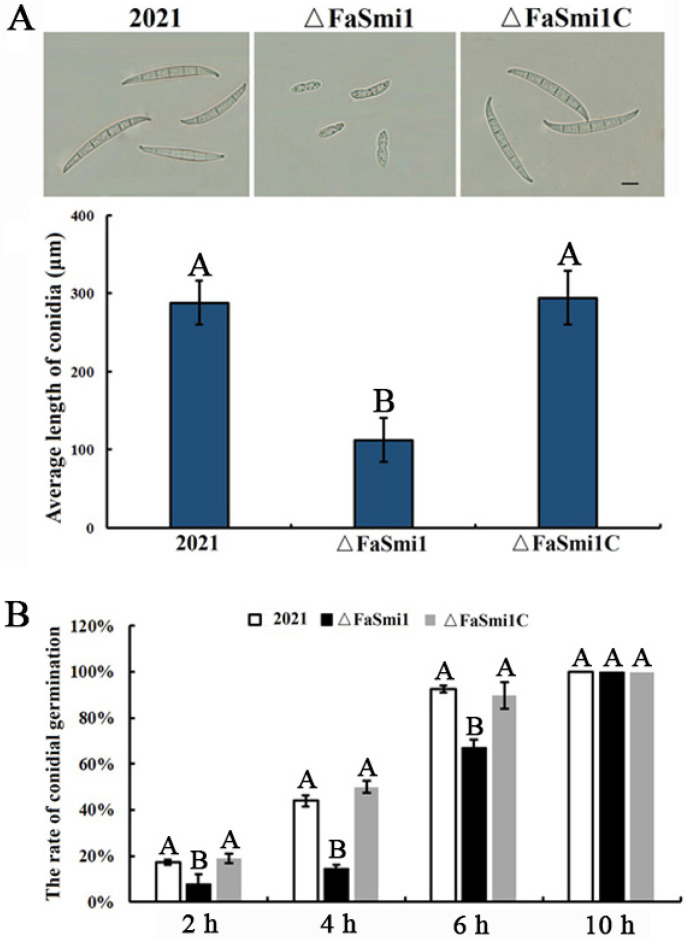
Effects of *FaSmi1* deletion on development and germination of conidia in *F. asiaticum*. (**A**) The conidia were isolated after mycelial plugs of the wild-type strain 2021, *FaSmi1* deletion mutant Δ*FaSmi1* and complemented strain (Δ*FaSmi1C*) were grown on MBB at 25 °C for 7 days. The conidia morphology of each strain was observed. Bar = 12 μm (up panel), the average length of conidia of each strain was measured. Bars denote standard errors from three experiments (down panel). Values on the bars followed by the same letter are not significantly different at *p* = 0.05. (**B**) Conidia of each strain were incubated on water–agar media at 25 °C. Conidial germination rates of each strain were measured at 2, 4, 6 and 10 h. Bars denote standard errors from three experiments. Values on the bars followed by the same letter are not significantly different at *p* = 0.05.

In the conidiation assay, Δ*FaSmi1* produced significantly fewer conidia compared to the 2021 and Δ*FaSmi1C* strains (Figure 2A; Table 1). Moreover, microscopically, we found that the conidia length of Δ*FaSmi1* was only about one third of the length of those of the 2021 and Δ*FaSmi1*C strains (Figure 2A). In a conidia germination assay, after incubation for 6 h, only 70% of the conidia of Δ*FaSmi1* were germinated, while almost all conidia of the 2021 and Δ*FaSmi1C* strains were germinated under the same conditions (Figure 2B). When the incubation time was extended to 10 h, all the conidia of Δ*FaSmi1* were germinated, indicating that the deletion of *FaSmi1* leads to defects in conidia germination. These results indicate that FaSmi1 plays a significant role in the conidial differentiation and germination in *F. asiaticum*. 

### 3.4. Involvement of FaSmi1 in Cell Wall Integrity

**Figure 3:**
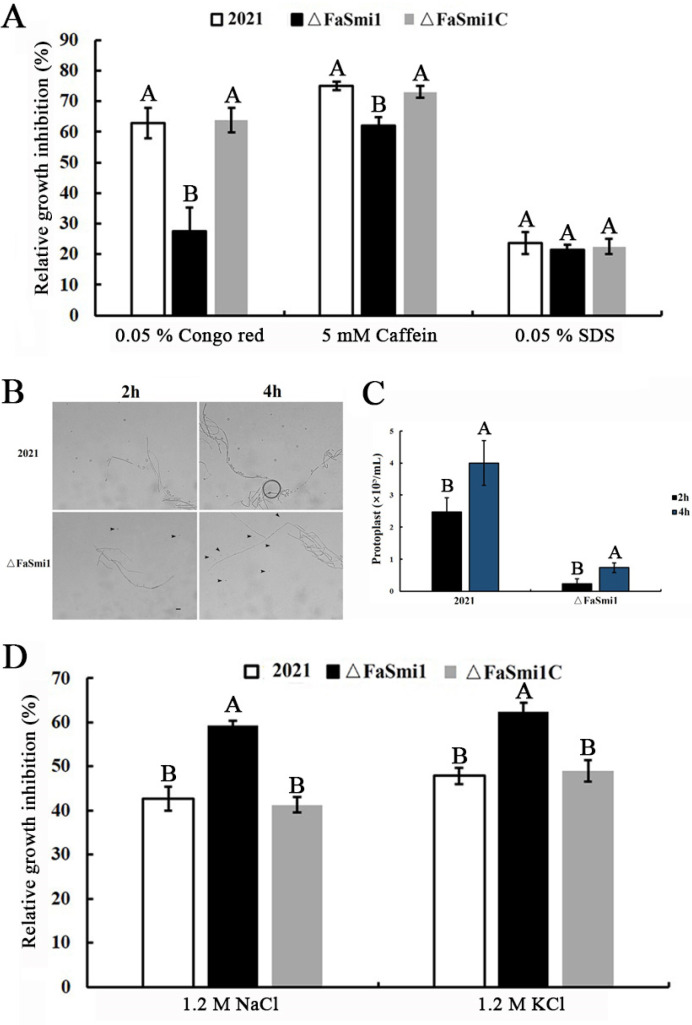
The deletion of *FaSmi1* is involved in response of *F. asiaticum* to cell wall inhibitors and osmotic stresses. (**A**) 2021, Δ*FaSmi1* and Δ*FaSmi1C* were grown on PDA medium amended with or without different stress factors at the indicated concentration. Inhibition of mycelial growth was determined by comparison of growth on the control treatment PDA. (**B**) Comparison of protoplasts released from mycelia among 2021 and Δ*FaSmi1* after incubation for 45 min in 1.5% lyase buffer at 30 °C. The protoplasts were indicated by the arrows. (**C**) The number of protoplasts released from each strain on lyase buffer were analyzed. Bars in each column denote the standard errors of three experiments. Values on the bars followed by the same letter are not significantly different at *p* = 0.05. (**D**) Inhibition rates of mycelial growth were analyzed after each strain was incubated for 3 days on PDA supplement with 1.2 M NaCl and 1.2 M KCl. Bars in each column denote the standard errors of three experiments. Values on the bars followed by the same letter are not significantly different at *p* = 0.05.

The previous research showed that Smi1 is involved in the cell wall formation and osmotic stress sensitivity of *S. cerevisiae*. To explore the role of FaSmi1 in cell wall integrity, we analyzed the sensitivity of Δ*FaSmi1* to the cell wall-damaging agents, Congo red (0.05%) and caffeine (5 mM). The results show that Δ*FaSmi1* exhibits decreased sensitivity to Congo red and caffeine compared to strains 2021 and Δ*FaSmi1C* (Figure 3A). Moreover, the number of protoplasts released from Δ*FaSmi1* after mycelia incubation at 30 °C for 2 h and 4 h in lyase buffer was obviously lower than that from the 2021 and Δ*FaSmi1*C strains (Figure 3B,C). These results suggest that FaSmi1 is required for the cell wall integrity of *F. asiaticum.*

### 3.5. Involvement of FaSmi1 in Osmotic and Oxidative Stresses Sensitivity

**Figure 4:**
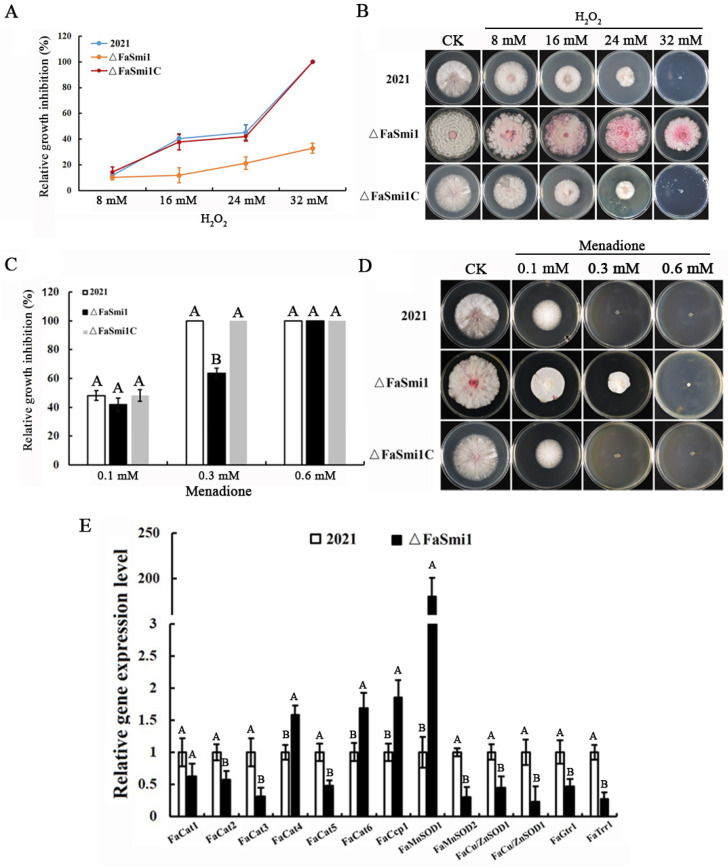
Sensitivity of 2021, Δ*FaSmi1* and Δ*FaSmi1C* to oxidation stress. (**A**) Each strain was incubated on PDA supplement with different concentration H_2_O_2_. When the colony diameter of the control treatment was greater than 6 cm, mycelial growth inhibition rate of each strain was analyzed. Bars denote standard deviation from three experiments. Values on the bars followed by the same letter are not significantly different at *p* = 0.05. (**B**) Colony morphology of each stain grown on PDA medium modified with different concentration H_2_O_2_. (**C**) Each strain was incubated on PDA supplement with different concentrations of menadione. When the colony diameter of the control treatment was greater than 6 cm, mycelial growth inhibition rates of each strain were analyzed. Bars denote standard deviation from three experiments. Values on the bars followed by the same letter are not significantly different at *p* = 0.05. (**D**) Colony morphology of each strain grown on PDA medium modified with different concentration menadione. (**E**) Relative expression levels of antioxidant system-related genes in 2021 and Δ*FaSmi1*. Line bars in each column denote standard errors of three repeated experiments. Values on the bars followed by the same letter are not significantly different at *p* = 0.05.

To test the sensitivity to osmotic stress, each strain was incubated on CM medium supplemented with the osmotic stress generator 1.2 M NaCl or 1.2 M KCl. The results show that the relative growth inhibition of Δ*FaSmi1* was higher compared to that of the 2021 and Δ*FaSmi1C* strains (Figure 3D). To test the role of FaSmi1 in oxidative stress responses, the mycelia growth inhibition rate of each strain was analyzed on CM plates modified with oxidative stress generators, including H_2_O_2_ and menadione. The results show that different concentrations of H_2_O_2_ and menadione inhibited Δ*FaSmi1* mycelial growth to a lower extent than that on 2021 and Δ*FaSmi1C* (Figure 4A–D). Additionally, qRT-PCR analysis showed that, compared to 2021 and Δ*FaSmi1C*, the expression level of the four genes associated with antioxidant responses were significantly up-regulated in Δ*FaSmi1*, especially the superoxide dismutase gene, *FaMnSOD1* (Figure 4E). These results indicate that FaSmi1 is involved in the osmotic and oxidative stress response. 

### 3.6. Effect of FaSmi1 on the Virulence and DON Production of F. asiaticum

**Figure 5:**
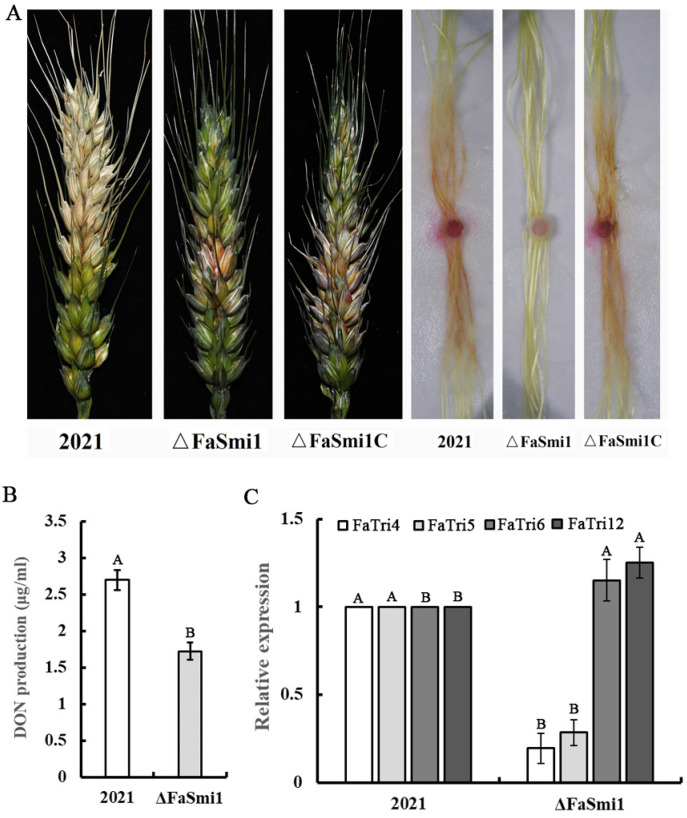
Effects of FaSmi1 on virulence and DON production of *F. asiaticum*. (**A**) Development of symptoms on flowering wheat heads and corn stigmas. Wheat heads were photographed after 2 weeks inoculated with a conidial suspension. Corn stigmas on filter papers were inoculated with brick-shaped mycelial plugs, and their pathogenic performance was examined after 3 days. (**B**) DON production of 2021 and Δ*FaSmi1* were measured after incubation in LTB for 7 days. Line bars in each column denote standard errors of three repeated experiments. Values on the bars followed by the same letter are not significantly different at *p* = 0.05. (**C**) The expression level of DON production-associated genes *FaTri4*, *FaTir5*, *FaTri6* and *FaTri12* were analyzed in 2021 and Δ*FaSmi1* after incubation in LTB for 2 days. Line bars in each column denote standard errors of three repeated experiments. Values on the bars followed by the same letter are not significantly different at *p* = 0.05.

To explore the role of FaSmi1 in the virulence of *F. asiaticum*, we analyzed the infection ability of each strain on wheat spikes via inoculating conidial suspensions into flowering wheat heads [25]. After being inoculated for 15 days, the 2021 and Δ*FaSmi1C* had infected and caused typical blight symptoms on inoculated spikelets, the lesion area had expanded over 50% of the whole wheat heads, while, under the same conditions, the Δ*FaSmi1* only infected spikelets at inoculation points, and the lesion cannot expand on the wheat heads (Figure 5A; Table 1). In addition, after inoculation for 3 days with brick-shaped mycelial plugs, when the virulence of each strain was analyzed on corn stigmas, the Δ*FaSmi1* failed to infect corn stigmas; however, the 2021 and Δ*FaSmi1C* had colonized and expanded on the corn stigmas (Figure 5A). In the DON production assay, the DON production of the *FaSmi1* deletion mutant was lower than that of 2021 (Figure 5B). In addition, compared with the parental strain, the expression level of DON synthesis-associated genes *FaTri4* and *FaTri5* was significantly downregulated in Δ*FaSmi1* (Figure 5C). The results indicate that *FaSmi1* is required for full virulence and DON synthesis in *F. asiaticum*. 

## 4. Discussion

In budding yeast, Smi1 has been reported to be a transcriptional regulator of gene expression, affecting cell wall biosynthesis and maintenance [28,29]. However, the investigation of its function was not detailed. Smi1 has 533 genetic and 42 physical interaction partners, which are involved in various cellular processes, including cell wall biosynthesis and maintenance, cell cycle, metabolism and osmoregulation [6,7,10,30,31]. Besides maintaining cell wall integrity, Smil probably plays important roles in various cellular processes. The functions of Smi1 were mainly reported in *S. cerevisiae*. They were not explored in plant pathogenic fungi. In our study, mycelial growth was obviously decreased in the *FaSmi1* deletion mutant, and spores of the *FaSmi1* deletion mutant were malformed compared to the wild-type and complemented strain (Figure 2). The Smi1 deletion mutant of *S. cerevisiae* showed increased sensitivity to lysis, Congo red, caffeine and SDS [29]. In this study, we found that the sensitivity of Δ*FaSmi1* to lysis, Congo red and caffeine was increased. The results indicate that the function of Smi1 on cell wall maintenance may be conserved among different fungi. In addition, we also found that the *FaSmi1* deletion mutant exhibits increased tolerance to oxidative stress generated by H_2_O_2_ and menadione (Figure 4), and the expression of the catalase genes *FaCat6* and *FaCcp1* and the superoxide dismutase gene *FaMnSOD1* was obviously upregulated in Δ*FaSmi1*. Moreover, the previous study showed that Smi1 is involved in stress tolerance by affecting the expression level of associated genes [32]. Based on these results, we inferred that the deletion of FaSmi1 leads to upregulation of the oxidative balance-associated genes *FaCat6*, *FaCcp1* and *FaMnSOD1*, which confers the tolerance ability of the FaSmi1 deletion mutant to oxidative stress. 

The DON production is an essential factor in the virulence of *F. asiaticum* that was influenced by various factors; ROS is a key enhancement factor for DON production [33,34]. Moreover, the expression of trichothecene cluster genes is essential for DON production [35,36]; Smi1 regulates metabolic process by affecting transcription-associated genes [32]. In this study, compared with wild-type and complement strains, in the *FaSmi1* deletion mutant, the trichothecene accumulation was obviously decreased (Figure 5B). Moreover, the expression of the catalase genes *FaCat6* and *FaCcp1* and the superoxide dismutase gene *FaMnSOD1* was upregulated (Figure 4D); in addition, the expression of DON synthesis-associated genes *FaTri4* and *FaTir5* in Δ*FaSmi1* was downregulated (Figure 5C). The results indicate that FaSmi1 involved in the regulation of DON may be associated to ROS balance. Taken together, we inferred that the deletion of FaSmi1 induces the expression level of *FaCat6*, *FaCcp1* and *FaMnSOD1*, which was obviously upregulated, leading to a decreased ROS accumulation level. The lower level of ROS is responsible for the downregulated expression of *FaTri4* and *FaTir5*, resulting in decreased DON production in *F. asiaticum* (Figure 4 and Figure 5). The previous study showed that nutrient sources, light, pH and signal transduction pathways are important regulation factors for DON production [36]. In our study, the results show that the cell wall integrity pathway core protein FaSmi1 regulates DON production by affecting the expression gene in the ROS metabolism system. Our data indicate that the ROS level may be another significant regulation factor for DON production in fungi.

In the virulence assay, we found that only Δ*FaSmi1* can infect the incubation site and loses the ability of extension in wheat ears, indicating the *FaSmil* is essential for the full virulence of *F. asiaticum* (Figure 5A). Based on our results, we considered that many factors are involved in virulence defects of the *FaSmi1* deletion mutant. First, the mycelial growth rate of Δ*FaSmi1* was obviously decreased (Figure 1A). Second, the *FaSmi1* deletion mutant exhibited a significant defect on spore germination (Figure 2A). Third, the DON production of Δ*FaSmi1* was obviously reduced (Figure 5C). Therefore, we concluded that virulence defects in the *FaSmi1* deletion mutant are closely associated with the defects on mycelial growth, spore germination and DON production. Taken together, our results indicate that FaSmi1 is essential for the vegetative development, asexual reproduction and virulence of *F. asiaticum*. Therefore, we thought that the FaSmi1 protein could be considered as a target for designing a new type of fungicide to control FHB caused by *F. asiaticum*.

## Figures and Tables

**Table 1:** Effect of *FaSmi1* on mycelial growth, conidiation and virulence of *F. asiaticum*.

Strains	Growth Rate on Three Media (mm/day) ^a^			Conidiation ^b^ (×10^5^ mL)	Percentage of Diseased Spikelets ^c^
	PSA	CM	MM		
2021	26.9 ± 0.3 ^A^	23.9 ± 0.7 ^A^	20.4 ± 0.3 ^A^	1.6 ± 0.1 ^A^	25.2 ± 3.5 ^A^
Δ*FaSmi1*	7.0 ± 0.2 ^B^	5.9 ± 0.2 ^B^	3.4 ± 0.6 ^B^	0.3 ± 0.3 ^B^	5.0 ± 2.4 ^B^
Δ*FaSmi1C*	27.9 ± 0.2 ^A^	25.1 ± 0.7 ^A^	21.1 ± 0.3 ^A^	1.5 ± 0.2 ^A^	24.4 ± 4.3 ^A^

^a^ Mycelial linear growth rate was tested on PSA plate. The radial growth of each strain was measured after 3 days at 25 °C. Mean and standard deviations were calculated with results from three replicates. ^b^ Sporulation assay was conducted in MBB. ^c^ The length of brown lesions on diseased stems 7 days post inoculation. Ten coleoptiles were inoculated for each. Different uppercase letters are used to mark statistically significant differences between strains (*p* < 0.05).

## Data Availability

Not applicable.

## Supplementary Materials

The following supporting information can be downloaded at: https://www.mdpi.com/article/10.3390/jof8111189/s1, Figure S1: Protein sequence of the selected fungal FaSmi1 orthologs were aligned with the BioEdit 3 software. Figure S2: The generation strategy and confirmation of FaSim1 deletion mutant and complement strains. Table S1: Primer used in this study.
