# Supplementary material for: FaSmi1 Is Essential for the Vegetative Development, Asexual Reproduction, DON Production and Virulence of Fusarium asiaticum
Source: J Fungi (Basel). 2022 Nov 11;8(11):1189. doi: 10.3390/jof8111189 (PMC9697231; doi:10.3390/jof8111189)
Supplement: Supplementary file 1 [file jof-08-01189-s001.zip › jof-1963563-supplementary.pdf]

Figure S1

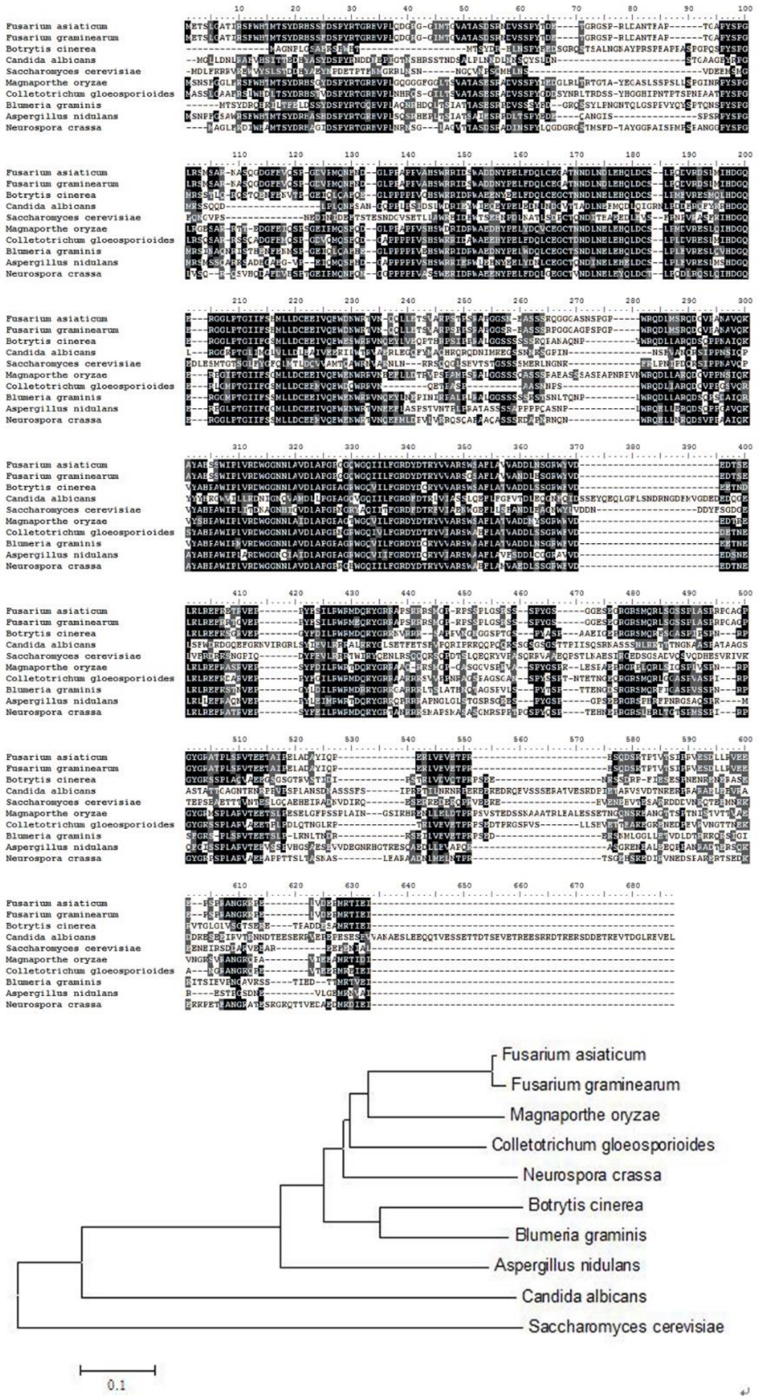

Figure S1. Protein sequence of the selected fungal FaSmi1 orthologs were aligned with the BioEdit 3 software. Identical amino acids are highlighted in gray. The protein sources are *Botrytis cinerea* (XP_024553765.1), *Candida albicans* (KGU13037.1), *Saccharomyces cerevisiae* (NP_011745.1), *Magnaporthe oryzae* (XP_003719889.1), *Colletotrichum gloeosporioides* (XP_011326681.1), *Blumeria graminis* (CAD6501685.1), *Aspergillus nidulans* (XP_682115.1), *Neurospora crassa* (XP_961149.3). Numbers indicate the amino acid positions. hylogenetic tree generated by the neighbour-joining method with Mega 4.1 software on the basis of the deduced amino acid sequences of FaSmi1 from *Fusarium asiaticum* strain 2021 and those from other fungi.

Figure S2

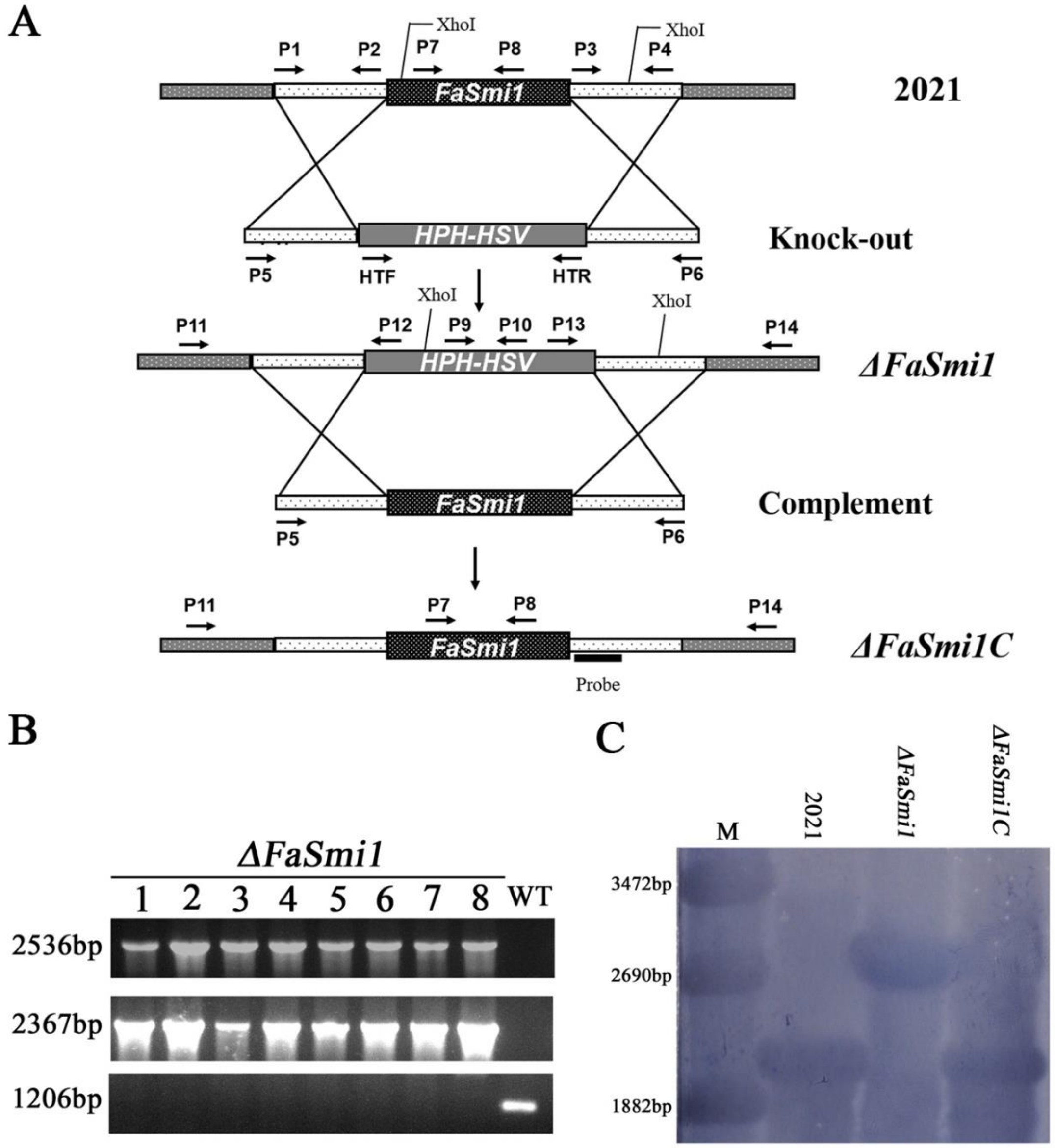

Figure S2 The generation strategy and confirmation of FaSim1 deletion mutant and complement strains.

A: FaSmi1 and hph-hsv fragments are denoted by large black and gray arrows, respectively. Annealing site of PCR primers are indicated with arrows (see Table S1 for primers sequences). B: PCR assay to screen ΔFaSmi1 transformants: PCR was performed with primer pair P11/P12; a 2139bp amplified fragment indicates *ΔFaSmi1* integration at the left junction. PCR was performed with primer pair P13/P14; a 2235bp fragment amplification indicates *ΔFaSmi1* integration at the right junction. PCR was performed with primer pair P7/P8 A 906bp amplification fragment indicates a WT (2021) locus. C: A 750bp FaSmi1 upstream fragment was used as a probe in Southern blot hybridization assay. Genomic DNA preparations from the wild-type strain (2021), the FaSmi1 deletion mutant (*ΔFaSmi1*) and the

complemented strain (*ΔFaSmi1C*) were digested with *XhoI*.

Table S1. Primer used in this study

Name	Sequence (5' to 3')	Relevant Characteristics
P1	AATAAATCTGTCAATCGGTG	Amplify the left homologous arm of *FaSmi1*
P2	CTTCAATATCATCTTCTGTTG GAGCTACAGAGGGAGA	
P3	AGACAATACCGGAAGGAAC GCGGTGTAGGGTAACTGA	Amplify the right homologous arm of *FaSmi1*
P4	CGAATACAACAGCCTCCT	
P5	CAGTCATCTGTAACCACCCA	Amplify the knockout vector of the *FaSmi1*
P6	TTTTCCAGAAGGATACCA	
P7	CGCCCCTTTACCCACATT	Amplify a partial fragment of the *FaSmi1*
P8	ACCCAGAACGAGCCACAA	
P9	GCAAACTGTGATGGACGACACC	Amplify a partial fragment of the *hph-hsv* gene
P10	ATCTCACCCTGGTCAAGGCGG	
P11	AATAAATCTGTCAATCGGTG	Confirm whether the *hph-hs*v gene homologously replaced *FaSmi1*
P12	ATCTCACCCTGGTCAAGGCGG	
P13	GCAAACTGTGATGGACGACACC	Confirm whether the *hph-hs*v gene homologously replaced *FaSmi1*
P14	CGAATACAACAGCCTCCT	
HTF	ACAGAAGATGATATTGAAGGAGC	Amplify the *hph-hsv* gene
HTR	TGTTCCTTCCGGTATTGTCTC	
FaSmi1-F	ATGGAGACCTCTCTCGGTGCGAC	Amplify the full cDNA or genomic sequence of the *FaSmi1*
FaSmi1-R	CAATGAAGACCATCGAGATTTAA	
Probe-F	GGATGAAACTTGGTGGTC	Amplify probe for the Southern blotting
Probe-R	TTGGTGAGGGCATTGTTA	
